# A longitudinal intervention study of the effects of increasing amount of meaningful writing across grades 1 and 2

**DOI:** 10.1007/s11145-023-10460-0

**Published:** 2023-06-08

**Authors:** Gustaf B. Skar, Steve Graham, Alan Huebner, Anne Holten Kvistad, Marita Byberg Johansen, Arne Johannes Aasen

**Affiliations:** 1 Norwegian Centre for Writing Education and Research, Department of Teacher Education, Norwegian University of Science and Technology, 7491 Trondheim, Norway; 2 University of Gothenburg, Gothenburg, Sweden; 3 Arizona State University, Tempe, USA; 4 University of Notre Dame, Notre Dame, USA

**Keywords:** Writing, Teaching writing, Increasing writing, Writing is caught approach

## Abstract

The current study examined the effectiveness of a *writing is caught* approach with young developing writers in Norway. This method is based on the premise that writing competence is acquired naturally through real use in meaningful contexts. Our longitudinal randomized control trial study tested this proposition by examining if increasing first grade students’ opportunities to write in various genres for different purposes and for a range of audiences over a two-year time period improved the quality of their writing, handwriting fluency, and attitude towards writing. The study included data from 942 students (50.1% girls) in 26 schools randomly assigned to the experimental treatment, and 743 students (50.6% girls) in 25 schools randomly assigned to the business-as-usual (BAU) control condition. Across Grades 1 and 2, experimental teachers were asked to supplement their typical writing instruction by implementing 40 writing activities designed to increase students’ purposeful writing. Increasing experimental students’ writing over the two-year period did not result in statistically detectable differences in the writing quality, handwriting fluency, and attitude towards writing of students in the experimental and BAU control conditions. These findings did not provide support for the effectiveness of the *writing is caught* approach. Implications for theory, research, and practiced are discussed.

Writing is complex. According to the Writer(s)-within-Community model (WWC; Graham, 2018a), it is fueled and molded by an author’s motives for composing, the utility and value ascribed to writing, presumed writing competence, writing identity, judgments about why writing is (or is not) successful, and beliefs about the community in which writing is produced. While composing, a writer draws upon multiple resources held in long-term memory including language and reading capabilities, knowledge about the writing topic, and specialized writing knowledge (e.g., schemas for producing text, knowledge of the purposes and structures of different kinds of writing, and knowledge about the conventions, norms, and purposes of writing in specific communities). In order to produce text, the writer employs multiple production processes including conceptualizing the writing task, accessing possible ideas for writing from long-term memory or external sources, translating these ideas into acceptable sentences, transcribing sentences into print or digital text, and reconceptualizing and transforming plans, ideas, and text. The writer initiates, orchestrates, and manages these cognitive and motivational resources as well as emotions, physiological responses, and personality traits through executive control processes involving goal setting, planning, monitoring, and corrections as needed. The mental work of writing is carried out in working memory where information is held and acted upon. Processing in working memory can draw upon reasoning, problem solving, decision making, analysis, and even intuition (Graham, 2021). The writer also employs attentional capabilities to choose what to attend to and what to ignore.

The cognitive capabilities and resources described above shape and constrain writing and a writer’s development (Graham, 2018b; Hayes, 2012), but these are not the only influential factors in play. The contexts or communities within which writing take place also shape and bound writing and its development (Graham, 2018a, 2023; Russell, 1997). For example, how writing is purposed, conceptualized, valued, and normed in a specific writing community influences the nature and form that writing takes. The writing tools, sanctioned and typical activities and actions for producing writing, the social (e.g., controlling or self-governing, cooperative or competitive, pleasant or unpleasant) and physical environment (physical, digital, or both), members’ familiarity and affiliation with the goals and operation of the writing community, and the enacted and collective history of said writing community all frame and constrain how writing is defined, produced, and learned. Moreover, each writing community is molded by cultural, social, institutional, political, and historical determinants as well as other social communities that its members have experienced (Graham, 2018b).

While schools are not the only community where young children write and learn to write (Rowe, 2023), these institutions are commonly tasked with teaching students how to write. As children begin school, they bring a variety of cognitive resources (e.g., knowledge of oral language) and capabilities (e.g., an increasing ability to maintain attention to specific tasks for a longer period of time) to this process, but there is much they must still learn, as described above, on the journey to increased competence and expertise as writers (Bazerman, 2018). According to the WWC model (Graham, 2018b), this development can be fostered by a variety of learning mechanisms. One mechanism is learning by doing which includes learning through participation in a writing community (e.g., as a child participates in a writing community, they learn its goals, identity, norms, action routines, identity, forms of reasoning, tool use, and so forth), learning as a consequence of writing (e.g., as students write they put into play various mental operations and behaviors and they acquire information on which are useful and not useful), and learning by expansion (e.g., as students read other people’s text, they can acquire important insight about how to write). A second mechanism in the WWC model is learning by observing others (e.g., students apply a writing procedure they saw another child apply or they observe how another person reacts to a particular text), whereas a third mechanism is learning from others (e.g., a teacher directly teaches students writing skills, strategies, or knowledge). A fourth mechanism is learning through deliberate agency (e.g., a writer makes a deliberate decision to become more skilled, apply what is learned in a previous situation to a new one, and building new ideas about writing within the context of old ones).

How these learning mechanisms are enacted with young writers depends upon the philosophical approach guiding writing instruction in schools. On one side of this philosophical debate is the *writing is taught approach* (Graham & Harris, 1997). With this approach, writing skills, knowledge, and processes are taught explicitly and systematically, and it is best exemplified by the learning from others mechanism in the WWC model, although learning by observing is often a part of such instruction (i.e., explicit instruction often involves teachers modeling how to apply specific writing skills and strategies, followed by guided practice in using them). Meta-analyses have demonstrated that explicitly teaching writing strategies, sentence construction skills, and spelling and handwriting is effective with young children (Graham & Santangelo, 2014; Graham et al., 2012; Koster et al., 2015; Santangelo & Graham, 2016).

On the other side of the philosophical debate, and the focus of the current study, is the *writing is caught* approach (Edelsky, 1990; Krashen, 1989). With this approach, writing is acquired naturally, much like learning to speak. Proponents argue that writing can be acquired through real use in meaningful contexts (Graham & Harris, 1997). Accordingly, writing proficiency can develop by providing students with plenty of opportunities to write and read for real purposes, assuming that much of what young children need to learn about writing can be “caught” by immersing them in a literacy rich environment. Teachers using such an approach also provide plenty of opportunities for students to share and display their writing as well as work together. Instruction is provided by capitalizing on teachable moments as the need arises. This can include modeling one or more aspects of writing. The writing is caught approach applies multiple learning mechanisms specified in the WWC model (Graham, 2018b) including learning through participation, consequence, expansion, observation, and from others. In contrast to the writing is taught approach, it emphasizes incidental and informal methods of learning and is best exemplified by the whole language approach (Goodman, 1992) and the process approach to writing (Pritchard & Honeycutt, 2006). While a review by Graham and Harris (1994) revealed that whole language was not any more effective than skills-based instruction in improving elementary-grade students’ writing, a meta-analysis by Graham et al. (2012) found that the process approach to writing did enhance the writing of Grade 2 to 6 students.

Finally, it should be noted that there is some overlap in the WWC model between a writing is caught and a writing is taught approach (e.g., learning through observation), and the two approaches can serve a complementary role to each other (e.g., writing provides an opportunity for students to apply taught skills). Even so, the study reported here attempted to isolate the effects of a writing is caught approach to learning to write.

## Study purpose

The current study examined the effectiveness of a *writing is caught* approach with children beginning first grade. In this longitudinal randomized control trial, teachers in the experimental condition implemented a writing treatment with Norwegian children that embodied a basic tenet underlying naturalistic approaches to teaching writing: writing is acquired through real use in meaningful context (Edelsky., 1990; Krashen, 1989). More specifically, students were provided ample opportunities to write in multiple genres for different purposes (Berge et al., 2016), and the writing students did in class served a real function and involved communication with a reader or audience (e.g., the teacher, parents, peers, or one-self) (Skar et al., 2020a, b). Across a two-year span of time, experimental teachers supplemented their typical writing program by implementing 40 writing activities, 10 a semester. For these writing activities, students shared what they wrote with their teacher, peers, or other readers. In some instances, these activities included other elements of writing is caught approaches such as peers working together, reading as a source of inspiration, and teacher modeling, but the overwhelming emphasis was on increasing opportunities to write for meaningful purposes. This was consistent with recommendations from the What Works Clearinghouse Practice Guide for elementary writing instruction (Graham et al., 2016) that emphasized daily time to write and creating a community of writers. The approach to teaching writing tested in this experiment provided a relatively stringent test of the theoretical proposition that young students’ writing is enhanced by writing for meaningful purposes. In accordance with the goals of this supplemental writing program, it was referred to as Functional Writing in Primary Schools. Based on the Norwegian spelling of this title, the acronym FUS is used here to refer to it. The writing of students in FUS was compared to students in business-as-usual (BAU) classrooms.

An inspiration for the FUS treatment was another Norwegian writing intervention study conducted in the mid-2010s by Berge et al. (2019) with students in Grades 3 to 7. This study was designed to increase the volume of writing done by students and broaden the focus of school writing to encompass writing for communicative purposes across different genres. The teachers who delivered the experimental intervention used a theoretical model, the Wheel of Writing (“the WW”; Berge et al., 2016), as a basis for designing writing tasks. The WW included six purposes for writing (e.g., to organize knowledge, and to convince), and in keeping with the traditionally strong teacher autonomy in Norway, the writing treatment was not standardized beyond teachers using the WW as a guide for writing tasks. Students (*n* = 153) received the WW intervention for two full school years and were compared to students from four control schools (*n* = 112). Students who received the WW treatment in Grades 3and 4 evidenced statistically larger improvement in the quality of their text than their corresponding peers in the control condition (*d* = 0.57), but there were no statistically detectable differences between experimental and control students in later grades. Like Berge et al. (2019), our study was designed to respect Norwegian teachers’ autonomy, as teachers developed their own writing activities based on guidelines provided by our research team and one in five lessons provided teachers with choice on the type of writing activity designed.

The present study extended research examining the effects of increasing writing in three important ways. One, prior research testing the effects of increased writing has involved elementary-grade students in Grades 3 and above (Berge et al., 2019; Gomez et al., 1996; Peters, 1991; Raphael et al., 1986; Soundy, 1987; Wienke, 1981), and it has produced small but statistically significant effects (average weighted effect of 0.30; Graham et al., 2012). We extended this prior research by examining if increasing students’ writing in a meaningful context was effective with even younger writers. We selected Grade 1 as our starting point because the Norwegian national curriculum has no “competency aims” for teaching writing at this grade. Several survey studies also found that students in Grade 1 spend little time writing at school (Graham et al., 2021; Håland et al., 2019). In addition, conducting this study with Grade 1 students in contrast to older students was advantageous because early exposure to a writing is caught approach should have an even greater impact on students’ writing growth than later implementation because students who are just learning to write are more positive about writing than older students (Graham, 2006), providing a more conducive situation for testing our hypotheses. Thus, first-grade provided us with an excellent opportunity to determine what happens when a supplemental writing program is implemented that is designed to disrupt BAU by significantly increasing the amount of purposeful writing students do.

Two, this study extended prior writing research by examining the effects of increased writing on a wider array of writing outcomes. Not only did we examine if writing quality improved, but we assessed the effects of FUS on handwriting fluency and students’ attitudes towards writing. Both of these outcomes are theoretically important to young writers’ development. According to the WWC model (Graham, 2018a), the production processes applied by writers requires conscious attention and cognitive resources. Young writers need to master transcription skills (e.g., handwriting) as early as possible because they can interfere with the execution of other production processes, such as content generation. It is important, therefore, to examine if increasing the amount of meaningful writing increases these students’ handwriting fluency.

The WWC model (Graham, 2018a) also posits that students’ motivations for writing, including their attitude towards writing, fuels writing effort and provides impetus for them to draw upon available cognitive resources. However, as students move from grade to grade, some studies have documented a decline in students’ motivations (Ekholm et al., 2018). As a result, it is important to track what happens to students’ attitude towards writing in response to specific writing treatments over time.

Three, this study extended most prior research on the effects of increased writing by conducting a two-year longitudinal investigation. Such a study provided ample time for the tested writing treatment to manifest changes in students’ writing outcomes. While Berge et al. (2019) also conducted a two-year study to test the effects of extra writing, their study was smaller in scope (24 schools vs 51 schools), involved older students, and included only assessed writing quality.

### Research question and predictions

This study answered the following question: Does a writing program designed to increase the amount of meaningful writing done by students in both first and second grade result in improved writing quality, faster handwriting fluency, and more positive attitudes toward writing?

We predicted that the FUS writing treatment would have a positive impact on the quality of students’ writing. Prior writing intervention research demonstrated that increasing the amount of writing done by elementary-grade students can improve writing quality (e.g., Berge et al., 2019; Graham et al., 2012; Peters, 1991; Raphael et al., 1986; Soundy, 1987; Wienke, 1981), whereas multiple learning mechanisms specified in the WWC model (Graham, 2018b), including learning by doing and learning as a consequence of writing, support the possibility of such effects.

We further expected that students in the FUS program would develop more fluent handwriting than those in the BAU control condition. We based this prediction on the common assumption that the young children’s handwriting fluency increases the more they write (Graham, 1999), and the FUS program is designed to increase the writing opportunities afforded to students who receive this treatment. We also predicted that FUS students’ attitude towards writing would be more positive than the attitude expressed by BAU control students, as the former would engage in more writing opportunities that were meaningful than the latter.

## Method

### Power analysis

As reported in a published research protocol for the project (Skar et al., 2020a, b), a pre-study power analysis estimate indicated that 60 schools would be needed in order to obtain an effect of 0.25 standard deviation for the writing treatment tested in this study, with an average of three classes per school and 16 students per class. Collectively, 61 Norwegian schools with first grade classrooms agreed to participate in the present investigation, with 31 of them randomly assigned to the FUS writing treatment and 30 to the BAU control condition.

### School recruitment

To recruit schools for participation in this study, the research team approached the education director of four municipalities in Norway (two large and two small municipalities) and presented the project to them. Each education director agreed to help the research team in the recruitment of schools. At the time of the investigation, there were 423 municipalities in Norway. Approximately 76% of students in Norway are served in large municipalities, 18% in average municipalities, and 6% in small municipalities. Consequently, the four municipalities in this study were representative of municipalities attended by 82% of Norwegian students.

To facilitate the recruitment process, the education directors of these four municipalities allowed the research team to present the project at meetings for school principals. School principals were informed of the planned content of the project, and that 50% of the schools would be randomly assigned to the treatment condition, while the other half would be assigned to a BAU control group. They were further informed that once the study was completed, schools in the control group would receive the FUS writing treatment. After these meetings, an email with detailed information about the project and a request to answer if the school was interested in participating in the project or not was sent to principals. Once 61 schools indicated they were interested in participating in the project, school recruitment ended.

Of the 61 schools that agreed to participate in the study, three schools opted out of the study after randomization, but prior to the administration of pretests. Two of these schools had been assigned to the control condition, and one had been assigned to treatment. At this point, there were 1678 students in the treatment classes and 1343 in the control classes. Parental consent was obtained for 1558 treatment students (92.4%) and 1139 control students (84.8%). Students from classes in another seven schools were excluded from the final sample of students in this study (four treatment and three control schools) because first grade teachers in those schools failed to administer one or more of the writing tasks at either pretest, posttest, or both. This left 942 first graders in the treatment condition in 26 schools and 743 students in the control condition in 25 schools who had completed all assessments at both testing times. These students formed the sample of participants for the current study. We decided to focus on the students from these 51 schools for two reasons. One, results for each writing outcome were based on the same students, making outcomes across measures comparable. Two, we ran all analyses with data from the 51 schools where all assessments were completed as well as the 58 schools where data for one or more tests were missing and the outcomes were the same.^1^ Only the outcomes for the 51 schools are reported here.

### Participants

Table 1 presents demographic information for the 1685 students in the 51 schools (125 classrooms) included in this investigation. Slightly more than one half of all students were boys (50.3%). Students were 73.77 months old (*SD* = 3.29) at the start of the study. According to students’ first grade teachers, 83.2% spoke Norwegian as their first language. Another 10.7% of students reportedly learned Norwegian and another language from birth, whereas 5.9% of students reportedly spoke a language other than Norwegian as their first language. There were no statistically detectable differences between students in the treatment and BAU control group in terms of chronological age, gender, and language with one exception. There were more students who spoke Norwegian as their first language in FUS than BAU control, *χ*^2^(1) = 23.22, *p* < 0.001.

**Table 1:** Characteristics of participating students

Variable	BAU control	Writing intervention	Total
Chronological age (months)	73.68 (*SD* = 3.26)	73.85 (*SD* = 3.30)	73.77 (*SD* = 3.29)
Girls	376 (50.6%)	472 (50.1%)	848 (50.3%)
Boys	367 (49.4%)	470 (49.1%)	837 (49.7%)
Norwegian first language	581 (78.2%)	821 (87.2%)	1402 (83.2%)
Norwegian not first language	51 (6.9%)	48 (5.1%)	99 (5.9%)
Norwegian and another language spoken from birth	111 (14.9%)	70 (7.4%)	181 (10.7%)

There were three participants for which the language background was not reported to the researchers

Students in the current study were generally representative of students in Norwegian schools broadly. The national percentage of girls in Norway is 48.9%, which compares favorably to 50.3% of girls in this investigation. While there are no public records indicating the percentage of students who speak a language other than Norwegian as their first language or that learned Norwegian and another language since birth, 7.9% of Norwegian students nationally receive extracurricular language instruction. This percentage is not much larger than the percent of students in this study identified by their teachers as not native speakers of Norwegian (5.9%).

The schools which taught participating students were also generally representative of schools in Norway. Scores for the 51 schools in this investigation on the obligatory National Test (NT) in reading, mathematics, and English were similar to national scores. The average NT result among the 51 schools was 51.0 (*SD* = 2.84), just slightly higher than the national average of 50 (*SD* = 10). The proportion of certified teachers (96.25%; *SD* = 4.24) in the 51 schools was close to the national average of 95%, as was the average number of instructional hours divided by the number of students (“school hours” for short) (i.e.,). A higher number of school hours indicate more time spent on instructional activities per student. Schools in this study averaged 54.67 h (*SD* = 11.10), whereas the national average was 61 h. It should be noted, however, that the average school in the present study (418.12; *SD* = 181.81) was larger than the national average (M = 225, SD = 166).

In terms of the school level variables, there were no statistically detectable differences between the FUS and BAU control students in terms of school size (treatment *M* = 445.85, *SD* = 173.56; BAU control *M* = 389.28, *SD* = 189.17), proportion of certified teachers (treatment *M* = 97.26, *SD* = 3.13; BAU control *M* = 95.09, *SD* = 4.97), or school hours (treatment *M* = 53.46, *SD* = 12.61; BAU control *M* = 55.92, *SD* = 9.38). There was, however, a statistically detectable difference between NT scores of students in the FUS writing treatment and BAU control schools. NT scores for the schools of FUS writing treatment students were higher (*M* = 51.75; *SD* = 3.02) than BAU control schools (*M* = 50.19; *SD* = 2.43), *t*(47.1) = 2.02, *p* < 0.05.

### FUS writing treatment

#### General instructional procedures

The writing intervention was implemented for four semesters during two school years, between fall of 2019 and spring of 2021. For each semester, students participated in ten writing activities, that involved one or several lessons which were typically 45 min in duration. The activities in each writing lesson were intended to supplement teachers already occurring classroom writing instruction.

The intervention was delivered by students’ teachers for two reasons. First, the current project aimed to test the effectiveness of a prior Norwegian writing treatment (Berge et al., 2019), and it was believed that if teachers delivered the program to their students, it could be rolled out nationally in the event of a positive outcome. The logic was that if the FUS writing treatment was successful despite the noise inherent in a research design where hundreds of teachers were involved (i.e., 125), the program would possess enough positive qualities to make it eligible as a national strategy. The second factor leading us to opt for teacher delivery was the size of the study. There were not sufficient resources for researcher-delivery in all classes.

Professional development (PD) for the FUS writing treatment was provided through eight face-to-face meeting with the participating teachers. In each meeting, detailed instructions on how to deliver the designed writing activities were provided. Two PD sessions were provided in the Fall of 2019; two PDs occurred during the Spring of 2020; two more PDs were offered in the Fall of 2020, and the final two PDs occurred in the Spring of 2021. During 2019, PD sessions were held in person at meeting venues, with most of the remaining PD sessions held digitally due to the COVID-19-pandemic. In each meeting, teachers were introduced and reminded of the core principles of “functional writing” around which the FUS writing treatment was centered (Skar, Aasen, et al., 2020). They were also presented with a thorough explanation and discussion of the 10 instructional writing activities to be delivered between this and the next PD session.

Following each PD session and one week in advance of each writing activity, instructions were sent directly to teachers’ email-addresses in print versions, computer screen versions, and mobile phone versions. These instructions included eight elements, which are described in Table 2. To illustrate, the instruction for a specific writing activity first provided a short description of the writing activity. Next, the association between the activity and Norwegian national curricular goals was specified, followed by a description of the expected outcomes (learning objective) as well as materials needed to complete the writing activity (tools). The instructions then provided a step-by-step-guide for completing the writing activity. In keeping with the Norwegian emphasis on teacher autonomy, teachers were told to adjust the step-by-step guide so that the activities were appropriate for their class. Teachers were asked to decide whether to divide an activity into two adjacent lessons delivered on the same day or lessons delivered on separate days. Most activities (62.5%) were designed to be completed in a week, with close to a third of the activities (32.5%) spanning two weeks and two activities (0.5%) spanning three weeks. The reason for this was logistic: this was a way to accommodate face-to-face instruction in the teacher sessions mentioned above, and to accommodate for the fact that schools across Norway have breaks at different times of the year. The instructions for a writing activity sent to teachers also included possible issues teachers might need to address when implanting it in their classes as well as an extended guide that provided more information on implementing the activity.

**Table 2:** Instructions for writing activities sent to teachers

Section	Content
Writing activity (e.g., Descriptive)	A short summary of the instructional activity (e.g., describe a superhero)
Curricular goals	A description of how the activity aligns with the Norwegian national curriculum for writing
Learning objective	A description of what students are expected to learn and/or experience through the activity
Tools	A description of the tools needed to conduct the activity (e.g., “scissor”, “envelopes”)
Step-by-step guide to the activity	A short step-by-step guide about phases of the activity (e.g., “teacher introduces the writing task”, “students plan their texts”)
Writing task	A prompt-like statement to use when children start writing (e.g., “write a text to yourself where you imagine yourself as an adult”)
Aspects to be considered when planning the activity	Questions for the teacher to reflect upon (e.g., “would you like students to share their texts? How would you do that?”)
Extended guide	An extension of the step-by-step guide detailing how to conduct the activities divided into pre-, during- and post-student writing. This extended guide would, for example, explain what it would mean for a teacher to introduce the writing task. The extended guide also contained suggested wordings for different phases of the activity

#### Specific instructional procedures

The core principals underlying the FUS writing treatment was that (1) students develop as writers if they are given ample opportunities to write in different genres for different purposes, and (2) writing should serve a function and be authentic in the sense that it entails communication with a reader (e.g., the teacher, parents, peers, or one-self).

Thirty-one of the 40 writing activities in the FUS writing treatment involved writing in a specific genre. Argumentative writing occurred four times, descriptive writing 15 times, narrative writing nine times, and reflective writing three times. For seven of the writing activities, teachers chose the writing genre. This was again in keeping with the Norwegian teacher autonomy tradition. The remaining two writing activities focused on transcriptional skills (i.e., handwriting and spelling), providing teachers with activities focused on integrating the teaching of transcription skills as part of writing or through games.

Table 3 presents the 40 writing activities in the FUS writing curriculum. Each activity is numbered in order, and the Table indicates when an activity occurred, how many parts the step-by-step-guide described, the genre targeted, the name assigned to the writing activity, and a short description of it. A more detailed description of one activity is provided below. Extended descriptions of all activities are available by request to the first author.

**Table 3:** Writing activities included in the FUS writing treatment

Activity	Parts	Genre	Short name	Contents
Week 33–34, 2019	1	Transcription skills	Using letters in text composition	Teachers provide students with different writing activities where letters can be used
Week 37, 2019	1	Optional	Joint composition of text	Teachers lead students in a joint writing process regarding a common experience (e.g., a class excursion, an experiment)
Week 38, 2019	1	Optional	Role play	Teachers facilitate role play (e.g., hospital, grocery store), and students write to participate in activity (e.g., signs, price tags)
Week 39, 2019	1	Transcription skills	Alphabet games	Teachers arrange an alphabet game
Week 40–42, 2019	1	Optional	Create a writing assignment	Teachers plan a writing assignment for students
Week 43, 2019	1	Optional	Create a writing assignment	Teachers direct a writing assignment
Week 44, 2019	1	Descriptive	Science report about paper spinners	Teachers lead student through an experiment with paper spinners with subsequent student writing of a science report
Week 45, 2019	1	Descriptive	Describe a superhero	Students create a superhero and provide a written description of that hero
Week 46, 2019	1	Descriptive	Everyday routines	Students describe an everyday routine in five steps
Week 47, 2019	1	Descriptive	Letter to the kindergarten	Students write a letter to kindergarteners to inform about life at school
Week 2, 2020	1	Optional	Joint composition of text	Teachers lead students in a joint writing process regarding a common experience (e.g., a class excursion, an experiment)
Week 3, 2020	1	Narrative	Model text from picture book	Students write a multimodal text using a picture book as basis
Week 4, 2020	1	Reflective	Write about the first hundred days at school	Students write a text about their 100 first days at school
Week 5, 2020	1	Descriptive	Science report about water and cold	The teacher leads student through an experiment with freezing water with subsequent student writing of a science report
Week 6–7, 2020	1	Descriptive	Describe a toy	The students write a description of a toy, with fellow students guessing which toy that has been described
Week 9–10, 2020	1	Narrative	Literature as a starting point for writing	Teachers read a passage from the book "The Dragon Hunt"–using "narration cards"–and students continue the story aided by narration cards
Week 12–13, 2020	1	Descriptive	Wanted Sign	Teachers direct a role play where a "thief" would "steal" a school artifact (e.g., a guitar) in front of the student with subsequent student writing a wanted sign
Week 18–19, 2020	1	Narrative	To plan a story	Based on video clips shown by teachers, students plan a narrative
Week 20, 2020	1	Descriptive	Writing a letter to next year's first graders	Students write a letter to next year's first graders, telling them about school
Week 21, 2020	1	Descriptive	Witches Brew recipe	Students write a recipe for a witch brew
Week 37, 2020	1	Descriptive	Science report about evaporation	Teachers lead an experiment with wet socks and evaporation with students then writing of a science report
Week 38, 2020	1	Narrative	To recognize elements in a story	Students practice recognizing elements in a story using "narration cards"
Week 39, 2020	1	Narrative	Recognize the narrative structure	Students receive a story cut into pieces and arrange the pieces in correct order
Week 40–42, 2020	1	Narrative	To write and talk about stories	Teachers read a story to students, and students fill out a story planning template and then plan and write their own story
Week 43, 2020	1	Narrative	To write a scary story (narration)	Teachers read a scary story to students and students write their own scary story
Week 44–45, 2020	1	Argumentative	Arguing for or against a topic	Students write an argumentative text for or against a topic that they are engaged in
Week 46–47, 2020	1	Optional	Create a writing assignment	Teachers plan and conduct a writing assignment
Week 48, 2020	1	Descriptive	Babysitter instruction	Students write instructions to a real or fictional babysitter
Week 49, 2020	1	Descriptive	Descriptive text about the reindeer	Students write a descriptive text about reindeer
Week 50, 2020	1	Argumentative	Application letter to Santa Claus	Students pretend they are reindeers and write an application letter to Santa Claus
Week 3–4, 2021	1	Argumentative	Arguing for or against a topic	Students write an argumentative text for something they want
Week 5, 2021	1	Argumentative	Argumentative text about recess time	Students talk about the fictional student Kim's text and then write their own argumentative text supported by a mnemonic
Week 6–7, 2021	1	Optional	Role play: Newspaper	Teachers facilitate a newspaper role play, and students write to participate in the play (e.g., articles, advertisements)
Week 9–10, 2021	1	Reflective	Write to reflect	Students write a text where they reflect over something that they want to accomplish
Week 11–12, 2021	1	Descriptive	Write a letter to your future self	Students write a letter to their future self
Week 14, 2021	1	Descriptive	Describe and guess	Students write a description of a place in the school yard, with fellow students guessing what place is described
Week 15–16, 2021	1	Descriptive	Descriptive text about an animal	Students choose an animal and write an informational text about it
Week 17, 2021	1	Narrative	Replacing narrative elements to create a new story	Students replace narrative elements in a story they know in order to create a new story
Week 18, 2021	1	Narrative	Use knowledge about narratives	Students write a narrative
Week 19–20, 2021	1	Reflective	Reflect on Writing Development	Students write about what they have learned about writing since they started school

Activity 35, *Write a Letter to Your Future Self* (see Table 3) informed teachers “In this activity the students are to describe themselves as adults: *Who am I? Where do I live? What is my occupation?* …” The audience for this activity was described as the student’s future self, and students should write in a way that will make them want to read it as adults. The curricular goals associated with this activity was “to be able to describe orally and in writing” and “be able to write texts by hand and on a keyboard.” The learning objective was for students to plan and design a text about their future selves. The tools students would need were paper, pencil, and envelopes. The step-by-step-guide described six steps to be carried out by the teacher:

1. Introduce the writing task
2. Talk about the content. Use a mind map or starter sentences.
3. Ask students to plan the text
4. Ask students to write and illustrate their text
5. Ask students to put their text into an envelope and seal it
6. Send envelopes home, asking students’ guardians to store them safely

Questions or issues for teachers to reflect upon included: “How can you present this writing task in a way that makes the student understand that the recipient is the student’s future self?” and “Do you want students to share their texts with peers? How should that be organized?”. Finally, the extended guide provided elaborations for the six steps above. For example, the elaborated second point (talking about content) included the following: “Talk about the content: *Who are you? How large is your family? What’s the name of members of your family? What do you like to do? Do you have any hobbies? What does your house look like? What does your car look like?* …”. Teachers were encouraged to stress that you should dream and have high hopes for the future. The directions to teachers also indicated they could have students use a mind map to visualize themes arising from the classroom discussion about future selves, or develop starter sentences based on ideas generated by students. Finally, the extended guide indicated teachers may want students to work together to generate ideas for their letter.

#### Fidelity

To assess fidelity of implementation, we queried participating teachers. For each writing activity, they were asked to indicate, on a scale from 1 to 6, how satisfied they were with their implementation of the writing activity. The number 1 indicated “least satisfied possible,” and 6 indicated “most satisfied possible.” When responding to this Likert-scale for each of the 40 writing activities, some schools had a single teacher representative respond for all participating first-grade teachers. In other cases, each teacher in a school responded only for themselves. When this was the case, the ratings of the teachers in that school were averaged to yield a single score for each school. On average two teachers (*SD* = 0.8) from each school responded. Across all 40 activities, FUS teachers averaged a mean score of 4.80 (*SD* = 0.5), indicating they were satisfied with their implementation of FUS activities.

We also asked teachers to indicate on a Likert-type scale (six levels from Strongly disagree [coded as 1] to Strongly agree [coded as 6]) to what extent it was possible to implement FUS writing activities during the COVID-19-pandemic, and to assess the extent to which the FUS-project had a positive impact on students’ writing. On average, FUS teachers weakly agreed they had been able to implement FUS writing activities as planned despite the COVID-19-pandemic (*M* = 4.2, *SD* = 1.40). They more strongly agreed, however, the FUS program had a positive impact on students’ writing (*M* = 5.4, *SD* = 0.9). Unfortunately, we do not possess more precise estimates regarding the proportion of teachers who successfully implemented all 40 writing activities as intended. The average score of 4.2 on implementation despite the COVID-19 pandemic indicate that that remote instruction and other measures taken by the Norwegian government may have impacted negatively on teachers’ possibility to fully enact the FUS program. It is important to note that in a program as extensive as FUS, some variation in implementation is expected.

### BAU control

Teachers in the BAU control condition were directed to continue teaching writing as they normally did. While we did not collect data on writing practices in BAU classrooms, a recent national survey of how writing is taught in Grades 1 to 3 in Norway (Graham et al., 2021) provides a general picture of what such instruction might entail. Typically, students in these grades spend about 20 min a day writing text that is a paragraph or longer (there was no statistically detectable difference in writing time across the three grades). When writing, they mostly write about content and less frequently produce narrative, descriptive, or explanatory text. The typical Grades 1 to 3 teacher applies numerous instructional procedures across the school year to teach writing, support students as they write, provide students with writing feedback, and conference with students. They less commonly teach planning or revising, promote motivation for writing, or use evaluation data to adjust instruction.

### Writing measures

Writing performance of students was assessed with: purposeful writing tasks aimed at real audiences, a copying task, and a questionnaire. When scored, these tasks, respectively, provided measures of writing quality, handwriting fluency, and attitude towards writing.

#### Purposeful writing tasks: writing quality

##### Task

To elicit samples of students’ purposeful writing, teachers administered two writing tasks at pre- and posttest designed to assess the quality of students’ writing. These writing tasks and the procedures used to score them were developed as part of this study, and they were based on previous research by Berge et al. (2019) and Skar et al., (2022a, b, c) to design tasks that reliably and validly measured children’s ability to write text adapted to a specified audience (Berge et al., 2019; Skar et al., 2022a, b, c). Skar, Jolle, and Aasen (2020a, b) documented the alignment between the writing quality measure in this study and the Norwegian national curriculum.

One of the writing tasks administered at pretest asked students to write a letter to researchers at the first author’s Norwegian university. In the letter, they were to tell these researchers what they enjoyed doing during recess time. The other pretest writing task asked students to describe the lunch box of their dreams to someone who could not see or smell their lunch box. At posttest, students again wrote a letter to researchers telling them what they did during recess time, but they also wrote in response to a prompt that asked them to provide an account of what happened when they found a magic hat. We assessed the similarities between lunch box and magic hat to be acceptable, as they both asked the students to do recounts. We anticipated that children starting school would not be able to write the more elaborate recount required by magic hat. Each of these writing tasks followed a specified structure where the topic was introduced, the purpose of the task was described, and what to write about was discussed. This discussion was supported by a visual aid (e.g., a picture of children playing for the recess prompt; a picture of a lunch box with various food items in it for lunch box of their dreams prompt).

To ensure the writing tasks were administered in a standardized manner, teachers were provided with printed instructions and a video on how to present them. Students were given a whole period (i.e., 45 min.) for each writing task, as this follows normal procedures in Norway for conducting such tasks. Further, teachers were asked not to assist students with the writing of their text beyond the discussion that occurred when presenting the tasks.

##### Scoring

When students finished each writing task, their texts were sent to the first author’s university where they were stripped of identifying information. This included removing information about students’ names, school, age, gender and anything else that could be considered to inform a rater about the student having written the text. Each text was scored by trained raters who assessed each text on eight separate validated rating scales (Skar, Jølle, et al., 2020a, b; Skar et al., 2022a, b, c). This included audience awareness, organization, content relevance, vocabulary, sentence construction, spelling, legibility and punctuation. For each scale, raters assigned a value between 1 and 5, with 5 indicating most quality.

Each of the rating scales addressed an important attribute of writing. For example, audience awareness assessed the degree to which a text communicated with the reader. The rating scale for organization focused on the internal structure of the text at the macro-, meso-, and micro-level. Content relevance concerned the scope of a student’s text that presented relevant content with regards to the writing task. The vocabulary rating concentrated on the repertoire of words used in the text, whereas sentence construction involved syntactical variation and syntactic complexity. Spelling assessed the increasing mastery of correct spellings in students’ texts, and legibility the shape and legibility of letters, including if letters were reversed or not. Lastly, the rating for punctuation concerned the correct use of punctuation marks. Prior to this study, the eight ratings scales were validated (Skar, Jølle, & Aasen, 2020a, b), and they have been used to assess thousands of texts (Skar et al., 2022a, b, c). Please refer to Appendix A in Skar et al., (2022a, b, c) for descriptors for all eight scales.

Texts from pretest and posttest were rated separately. In all, 26 trained raters participated in the rating of pretest compositions and 24 trained raters participated in the rating of posttest papers. Ten raters participated in rating at both time points. Raters were seasoned academics with expertise in literacy as well as graduate students with specialization in the same area. With text produced at both pretest and posttest, raters were trained to use the eight rating scales. This included detailed instruction on how to use each ratings scale, and a training session in which raters practiced scoring compositions, followed by group discussions of assigned scores. We tested raters’ consistency in scoring text across time (pretest and posttest) by having raters at each time point score the same 50 texts. There was no statistically detectable difference in scores assigned by pretest and posttest raters, *F*(2, 147) = 0.17, *p* = 0.84. All compositions were scored independently by two raters. The “Rasch Reliability” which is analogues to Cronbach’s alpha at pretest was 0.94; it was 0.95 at posttest.

##### Many-facet Rasch model

All raw scores for ratings of students’ text were fitted to a many-facet Rasch measurement” (MFRM) model (Linacre, 2018a, b) to yield a single text quality score 50 texts were distributed to all raters and these texts were used as “linking devices” in the many-facet Rasch analysis. The MFRM software *Facets* outputs a “fair score,” which in this case represented a student’s average score (ranging from 1 to 5, with larger scores representing better quality text) across all texts/tasks, scales, and raters after controlling for difficulty of task, scale, and rater severity respectively. As a rule of thumb, data is said to fit the MFRM model if there are less than 1% of standardized residuals exceeding 3, and less than 5% of standardized residuals exceeding 2 (Eckes, 2011). At pretest, 2.78% of the standardized residuals were in the range of |2–3| and 1.47% exceeded |3|, meaning that there were a few standardized residuals with higher values than wished for. At posttest, 4.5% of the standardized residuals were in the range of |2–3| and 0.5% exceeded |3|. Considering the small deviation at pretest from the rule of thumb together with the high reliability we deemed the data to fit the MFRM model.

#### Copying task: handwriting fluency

To assess handwriting fluency, students copied a paragraph from the Group Diagnostic Reading and Aptitude and Achievement Tests (Monroe & Sherman, 1996). This task was used in a previous investigation assessing the handwriting fluency of Norwegian children in Grades 1 to 3 (Skar et al., 2022a, b, c), and it has been applied in prior investigations in other countries (e.g., Graham et al., 1997). With this task, students are prompted to quickly and as accurately as possible copy a paragraph of text in 1.5 min. The number of letters copied correctly was divided by 1.5 to compute number of letters written per minute. This served as an estimate of students’ handwriting fluency. Letters that were written correctly, but did not match the text in the paragraph, were not counted as correct, nor were incorrectly written letters (i.e., letters that were provided but did not match the letter in the paragraph) or skipped letters.

This copying task was administered by students’ teachers at pretest and posttest. Teachers used a video to introduce the task. The video informed students that the teacher would read a paragraph aloud, and they would copy as much of the text as possible during a 90 s interval. The video also informed students teachers would signal when to start and stop copying the paragraph.

Trained coders at the first author’s university scored the copied material produced by each student. To establish reliability 10% of the copied texts were double coded. The reliability was κ = 0.812, and the ICC was 0.99.

#### Questionnaire: attitude towards writing

After completing the purposeful writing tasks administered at pretest and posttest, teachers asked students to complete a questionnaire that included the following four items assessing attitude towards writing: “I liked the writing task,” “I am satisfied with my text,” “I am satisfied with my effort,” and “I like to write.” To rate each statement, students used a Likert-style star system: three stars represented most agreement (a score of 3), and one star represented least agreement (a score of 1). A student’s score for this measure was the average of the three items. An exploratory factor analysis of the four-item measure at pretest revealed it represented a single factor, accounting for 53.8% of the variance (coefficient alpha = 0.71).

### Procedures

Prior to the start of the study, the project was reviewed and approved by the Norwegian Centre for Research Data (Project No.: 848410). Following this approval, schools were contacted, and those that agreed to participate were randomly assigned to FUS or BAU control. All parents of first-grade students in participating schools were then contacted and informed about the project. Students only participated if they received active parental consent. Parent consent was provided for 89% percent of students. Students who were not consented in FUS schools still participated in the writing treatment because it was part of their regular classroom curriculum, but data on the effects of the treatment on the quality of their writing, handwriting fluency, or attitude towards writing was not collected by researchers.

Pretest measures were administered in August to September 2019, whereas posttests were administered May to June, 2021. Teachers in the experimental condition delivered the FUS writing treatment across a two-year period (Grades 1 and 2). Teachers in the BAU control condition continued teaching writing as they normally did, but they received instruction using the FUS curriculum once the study was completed.

### Analysis

The data collected for this study was hierarchical as students were nested within classrooms and classrooms were nested within schools.^2^ Consequently, we applied a three-level linear multilevel regression model (MLMs) for each assessed writing outcome. More specifically, for writing quality, handwriting fluency, and attitude towards writing, a linear MLM with no predictors (i.e., an unconditional model, or “null” model) was first fit to assess the correlation structure of the data by computing intraclass correlation coefficients (ICCs), and next a linear MLM including the variable of interest (FUS writing treatment vs BAU control) as well as the covariates (student gender, age, and language background as well as school-level covariates of national test results, number of students per school, proportion of certified teachers, and number of students per special education teacher) were used to test the “full” model. Fourteen of the participating students had missing school data, 23 students were missing information on their chronological age, and data on the language background of three students was not available. Given the small number of missing observations, mean imputation was used for the missing school and age data, and the missing language was set to “Norwegian”, the most common language background in the sample.

All MLMs were fit using the *lme4* package (Bates et al., 2015) in the *R* statistical software environment (R Core Team, 2022). Table 4 displays effect sizes for the models for each outcome. Three-level MLMs allow for two ICCs to be calculated, describing the correlation between students within a school in different classes and the correlation between students within a school in the same class. For example, the estimated correlation for handwriting fluency between two randomly selected students in the same school is 0.060, whereas the estimated correlation for handwriting fluency between two randomly selected students in the same classroom is 0.139. Writing quality exhibited the strongest correlations within schools and classes, and attitude towards writing had the lowest. In addition, Table 4 displays ${R}^{2}$, the amount of variation in the outcome variable explained by predictors, for the full model for each outcome. The model for writing quality explained just over 20% of the variation for that outcome, which was the largest ${R}^{2}$ among the full models for the three writing outcomes. Further, the effect size ${f}^{2}={R}^{2}/(1-{R}^{2})$ is given in the last row of Table 4. Lorah (2018) indicated effect sizes of 0.02, 0.15, and 0.35 represent small, medium, and large effects, respectively. The effect sizes for the full models for handwriting fluency and writing quality can be considered as medium, while the effect size for motivation is between small and medium.

**Table 4:** Variance components and effect sizes for models for each writing outcome

Quantity	Writing quality	Handwriting fluency	Attitude
${\sigma }_{e}^{2}$(student variance)	0.242	113.355	0.196
${\sigma }_{c}^{2}$(class variance)	0.041	10.511	0.008
${\sigma }_{s}^{2}$(school variance)	0.026	7.885	0.009
ICC (class)	0.217	0.139	0.080
ICC (school)	0.084	0.060	0.042
${R}^{2}$(full model)	0.208	0.138	0.106
${f}^{2}$(full model)	0.262	0.160	0.118

*Attitude* Attitude towards writing

## Results

Tables 5, 6, 7 provides means and standard deviations for writing quality, handwriting fluency, and attitude towards writing, respectively, by treatment condition (FUS and BAU control) for all students in each condition, girls and boys, and student language status (L1, L2, and bilingual). Regression coefficients and corresponding *p*-values for the full model for each writing outcome are displayed in Table 8. The parameters estimated included regression coefficients for treatment (FUS vs BAU control), student covariates (gender, age, language status, and pretest scores), and school covariates (national test scores for school, size of school, proportion of certified teachers, number of students per special education teachers, and number of instructional hours). For the three analyses, the pretest score (e.g., pretest writing quality) corresponded to the writing outcome of interest (e.g., posttest writing quality).

**Table 5:** Means and standard deviations for writing quality at pretest and posttest

		Pretest writing quality		Posttest writing quality		Effect size
		*M*	*SD*	*M*	*SD*	Cohen’s *d*
FUS	Girls	1.37	0.44	3.43	0.51	4.27
	Boys	1.26	0.37	3.10	0.57	5.02
	L1	1.32	0.41	3.26	0.57	4.70
	L2	1.22	0.29	3.22	0.60	6.94
	Bilingual	1.33	0.39	3.40	0.50	5.26
	All Ss	1.31	0.41	3.26	0.56	4.80
BAU control	Girls	1.32	0.37	3.28	0.49	5.33
	Boys	1.25	0.36	2.99	0.54	4.89
	L1	1.31	0.37	3.17	0.53	4.98
	L2	1.10	0.20	3.03	0.41	9.58
	Bilingual	1.21	0.35	3.05	0.59	5.32
	All Ss	1.29	0.36	3.14	0.54	5.10

*FUS* FUS writing treatment; *L1* Students who spoke Norwegian as their first language; *L2* Students who spoke a language other than Norwegian as their first language; *Bilingual* Students who learned Norwegian and another language from birth; *Ss* Students

**Table 6:** Means and standard deviations for handwriting fluency at pretest and posttest

		Pretest handwriting fluency		Posttest handwriting fluency		Effect size
		*M*	*SD*	*M*	*SD*	Cohen’s *d*
FUS	Girls	6.67	3.71	29.83	11.85	6.24
	Boys	5.22	3.10	23.71	10.72	5.96
	L1	5.94	3.46	26.41	11.40	5.92
	L2	5.01	2.66	29.21	13.88	9.09
	Bilingual	6.58	4.26	29.43	13.22	5.37
	All Ss	5.94	3.50	26.77	11.71	5.96
BAU control	Girls	6.30	3.45	27.44	11.64	6.12
	Boys	5.30	2.93	22.56	9.59	5.89
	L1	5.85	3.29	25.41	11.18	5.94
	L2	4.30	2.64	22.78	9.17	7.01
	Bilingual	6.31	3.06	24.12	10.29	5.82
	All Ss	5.81	3.24	25.03	10.94	5.92

*FUS* FUS writing treatment; *L1* Students who spoke Norwegian as their first language; *L2* Students who spoke a language other than Norwegian as their first language; *Bilingual* Students who learned Norwegian and another language from birth; *Ss* Students

**Table 7:** Means and standard deviations for attitude towards writing at pretest and posttest

		Pretest attitude		Posttest attitude		Effect size
		*M*	*SD*	*M*	*SD*	Cohen’s *d*
FUS	Girls	2.66	0.45	2.63	0.38	− 0.07
	Boys	2.57	0.51	2.35	0.49	− 0.42
	L1	2.61	0.48	2.47	0.46	− 0.27
	L2	2.65	0.43	2.67	0.37	0.04
	Bilingual	2.64	0.48	2.56	0.43	− 0.17
	All Ss	2.61	0.48	2.49	0.45	− 0.26
BAU Control	Girls	2.60	0.49	2.54	0.41	− 0.14
	Boys	2.59	0.48	2.32	0.50	− 0.57
	L1	2.59	0.49	2.42	0.46	− 0.36
	L2	2.72	0.39	2.56	0.52	− 0.41
	Bilingual	2.56	0.48	2.41	0.50	− 0.30
	All Ss	2.60	0.48	2.43	0.47	− 0.35

*Attitude* Attitude towards writing; *FUS* FUS writing treatment; *L1* Students who spoke Norwegian as their first language; *L2* Students who spoke a language other than Norwegian as their first language; *Bilingual* Students who learned Norwegian and another language from birth; *Ss* Students

**Table 8:** Estimated regression coefficients, standard errors, and statistical significance for the full model for each writing outcome

	Outcome		
	Handwriting fluency	Writing quality	Attitude towards writing
Intercept	23.056*** (1.045)	3.042*** (0.045)	2.291*** (0.032)
Treatment	1.394 (1.388)	0.040 (0.060)	0.058 (0.041)
Gender (Girl)	4.414*** (0.482)	0.291*** (0.022)	0.245*** (0.021)
Age	0.280 (0.243)	0.033** (0.011)	− 0.021* (0.011)
Language: L2	− 0.378 (1.149)	− 0.075 (0.053)	0.148** (0.048)
Language: L1 + L2	− 0.0658 (0.854)	0.010 (0.040)	0.040 (0.036)
Outcome pretest	3.908*** (0.259)	0.162*** (0.012)	0.064*** (0.011)
NT	0.880 (0.798)	0.093* (0.034)	0.001 (0.023)
School size	− 0.196 (0.689)	− 0.003 (0.030)	0.006 (0.020)
PropCertif	− 0.290 (0.708)	0.012 (0.031)	0.031 (0.021)
StudSpecEd	0.080 (0.836)	− 0.078 (0.030)	0.013 (0.020)
Hours	0.057 (0.685)	− 0.014 (0.036)	0.019 (0.025)

For the Gender covariate, “Boy” was taken as the reference level, and thus the coefficient refers to the expected increase for Girls; for Language the reference was Norwegian as students’ native language; L2 = students with a language other than Norwegian as their native language; treatment contrasted FUS and BAU control; NT = national test; PropCertif = proportion of teachers certified; StudentSpecEd = number of students assigned to special education teachers; Hours = average number of school hours. Statistical significance is indicated such that “*”, “**”, and “***” denote $0.05<p-value\le 0.01$, $0.01<p-value\le 0.001$, and $p-value<0.001$, respectively

### Writing quality

As can be seen in Table 5, writing quality improved by about two points on the five-point scale for both the FUS treatment (mean improvement of 1.95) and the BAU control students (mean improvement of 1.85) over the course of the two-year experiment. For writing quality, the results of the three-level MLM revealed statistically significant coefficients for gender, age, and pretest writing quality scores. The 95% confidence interval for gender was 0.25–0.33, 0.01–0.06 for age, and 0.14–0.19 for pretest writing quality. The coefficient for treatment (FUS vs BAU control) did not result in a statistically detectable effect (confidence interval was − 0.07 to 0.15).

### Handwriting fluency

From the start of first grade to the end of second grade, handwriting fluency evidenced a 451% increase for FUS treatment students and a 431% increase for BAU control students (see Table 6). For handwriting fluency, the results of the three-level MLM revealed statistically significant coefficients for gender and pretest handwriting fluency scores. The 95% confidence interval for gender was 3.36–5.35 and 3.29–4.41 for pretest quality. The coefficient for treatment (FUS vs BAU control) did not result in a statistically detectable effect (confidence interval was − 1.19 to 3.94).

### Attitude towards writing

Attitude towards writing declined slightly for FUS (11% decline) and BAU control students (12% decline) from the beginning of first grade to the end of second grade (see Table 7). For attitude towards writing, the results of the three-level MLM revealed statistically significant coefficients for gender, age, language status, and pretest writing quality scores. The 95% confidence interval for gender was 0.20 to 0.29, − 0.04 to 0.00 for age, 0.06 to 0.25 for Norwegian as a second language (L2) compared to Norwegian as a first language (L1), and 0.04 to 0.09 for pretest attitude towards writing. The negative sign on the coefficient for age means that younger students tended to report more positive attitudes toward writing. The coefficient for treatment (FUS vs BAU control) did not result in a statistically detectable effect (confidence interval was − 0.02 to 0.13).

## Discussion

Despite the importance of writing to school, work, and everyday life, there is considerable disagreement about how writing is best acquired. Some scholars argue that the best way to promote students’ writing development is through a *writing is caught* approach (Edelsky, 1990; Krashen, 1989). This method to teaching writing assumes that writing competence is acquired naturally through real use in meaningful contexts (Graham & Harris, 1997). This includes providing students with plenty of opportunities to write for communicative purposes. The current longitudinal randomized control design study tested this proposition by examining if increasing first grade students’ opportunities to write in various genres for different purposes and a range of audiences over a two-year period of time would enhance the quality of their writing, handwriting fluency, and attitude towards writing in comparison to BAU control students.

### Increased opportunities to write did not enhance students’ writing

Contrary to our predictions, first-grade students in Norway who were provided with two-years of extra practice writing for communicative purposes through the FUS program did not produce qualitatively better text, evidence greater handwriting fluency, or exhibit more positive attitude than students who did not receive such supplemental instruction. Theoretically, we had assumed that increasing the amount of writing students did for communicative purposes would have a positive impact on writing because they would learn by doing and as a consequence of writing (Graham, 2018b). These findings did not provide support for the *writing is caught* approach with beginning writers and draws into question the power of the learning mechanisms underlying this approach.

The findings from this study stand in contrast to other investigations where increasing how much elementary-grade students wrote had a positive effect on the quality of the text they produced. Most of the prior studies with positive effects were conducted in the U.S. (Peters, 1991; Raphael et al., 1986; Soundy, 1987; Wienke, 1981). It is possible that contextual differences in schooling (e.g., teacher autonomy), students served (Norway is a less diverse country), and how writing was typically taught may have contributed to differences in the outcomes for this investigation and these prior studies. For instance, the FUS program involved supplemental writing which was added to Norwegian teachers typically writing practices. It is possible writing instructional practices in the U.S. and Norway are divergent enough they differentially impact the effects of increased writing. Future research needs to explore if types of writing instruction provided interacts with experimental methods to increase how much students write. It is also important to study if such interactions are further influenced by different approaches for increasing the amount of writing.

Another possible explanation for differences in outcomes between this and prior investigations (Peters, 1991; Raphael et al., 1986; Soundy, 1987; Wienke, 1981), including the Berge et al. (2019) study where increased writing enhanced the quality of Norwegian students’ text, concerns student grade-level. All of the previous investigations with positive outcomes involved elementary students in Grades 2 or higher. Students in the present study had just enrolled in Grade 1 at the start of the study. It is possible that students are not able to take advantage of a *writing is caught* approach until they are older. As a result, it is important to replicate the current study with first-grade students in different contexts to test whether increasing meaningful communicative writing activities beginning in Grade 1 and continuing through Grade 2 enhances young students’ writing. It should not be automatically assumed that increasing writing will lead to positive gains for at all grades or developmental-levels. For example, a meta-analysis of writing interventions with Grades 6 to 12 students (Graham et al., 2023) did not find that increased writing had a positive effect on writing. As a result, a writing is caught approach may only impact students’ writing growth at critical points in students’ development as writers. If findings from future research studies provide more definitive data on this issue, then it will be important to explore why this is the case. It must also be noted that differences in writing outcome for this investigation and the Norwegian study by Berge et al. (2019) may reflect more than grade-level differences. They may also reflect differences in how the experimental treatments were conceptualized and delivered. The current study was inspired by Berge et al. (2019), and in both studies teachers played a central and autonomous role in designing writing activities. It is possible that the extra writing activities in the earlier study were more effective than the ones in this investigation, because teachers in Berge et al. (2019) designed better writing activities. If this was the case, it only occurred for students in Grade 3, as increased writing did not improve the writing of older students in the earlier investigation.

While other explanations can be proposed for why increasing meaningful and communicative writing did not improve student outcomes in this study (e.g., large studies such as this one consistently yield null or small effects; Kraft, 2020; Slavin & Smith, 2009; teachers in the control condition were already doing considerable and meaningful writing with their students), ultimately it must be recognized that the findings from this investigation did not provide support for the *writing is caught* methods or the learning by doing or learning as a consequence of writing mechanisms (Graham, 2018b). This does not definitively mean the activities applied in this study had no effect. It is possible that our assessments did not adequately capture what students learned. Consequently, researchers may want to expand the types of assessments administered in future studies like this one. For example, students who participate in a supplemental program like FUS may develop a better understanding of how writing works in their classrooms (e.g., the goals, values, stance, and audience for writing in their class) and what constitutes good writing (e.g., norms and evaluative criteria for writing). It is also possible that increasing young students’ writing through the FUS program was not as effective as predicted because students did not spontaneously evaluate their actions when writing. Research is needed to determine if adding a metacognitive component to programs like FUS, where students make judgments about what aspects of the writing activity were useful and successful, leads to writing growth.

Finally, it is unclear how the Covid-19 pandemic influenced the impact of the FUS treatment. Teachers as a group slightly agreed that it had little effect, but this was not the judgment of all teachers. This must be taken into account when interpreting the outcomes.

### Implications for instruction

One implication from the current study for teaching writing is that increasing how much first-grade students write, even when such writing is communicative and provided over a two-year period, is not enough to ensure they make greater progress than peers who receive typical writing instruction. This is not to say that young students should not write for meaningful purposes and a real audience. In a recent meta-analysis with students in Grades 6 to 12, Graham and colleagues (2023), found that increasing how much students wrote did not improve their writing (effect size = 0.14), but when writing was part of a broader writing program where students did more than write, they evidenced statistically significant improvements in writing (effect size = 0.44). As a result, a writing program without student writing is not a sound proposition.

We further found that girls had higher scores than boys on all three writing outcomes: writing quality, handwriting fluency, and attitude towards writing. Similar gender effects have been noted in the U.S. (Ekhom et al., 2018; Reilley et al., 2019). This suggests that teachers may want to pay particular attention to the writing development of young boys to ensure they make adequate progress as writers. This same caution may also apply to the youngest students at the start of first grade, as older first grade students tended to be better writers in this investigation.

We further noticed a slight decline in attitude towards writing from the start of Grade 1 to the end of Grade 2 for both FUS and BAU control students. If such a decline continued across subsequent grades, it could have a negative impact on students’ engagement when writing and their willingness to write (Graham, 2022). It may be especially important for teachers to monitor such motivational changes over time, and put into place procedures that can lead to more positive beliefs (e.g., positive feedback, students working together, choice).

Lastly, Norwegian students’ handwriting fluency at the start of first grade was only six letters per minute and 26 letters per minute at the end of second grade. This is slower than the handwriting fluency scores of 19 letters per minute for Grade 1 and 34 letters per minute for Grade 2 students in the U.S. (Graham et al., 1998). Because handwriting fluency can interfere with other writing production processes such as sentence generation and planning, Norwegian teachers in Grades 1 and 2 may want to consider putting into place instruction designed to enhance this critical skill (see Graham, 1999).

### Limitations and conclusion

There are several limitations that must be considered when interpreting the outcomes in this investigation. First, we do not have a clear picture of how teachers in the BAU control condition taught writing. Previous survey studies conducted with Grades 1 and 2 Norwegian teachers found that students do relatively little writing in these grades (Graham et al., 2021; Håland et al., 2019). This may not have been the case for control teachers in the current study. Second, teacher fidelity was assessed using self-report measures. This provided a limited estimate of how well teachers implemented the FUS program. Moreover, it is not impossible that the COVID-19 pandemic had a negative impact on teachers' implementation of the program. However, we were unable to obtain specific data on how the pandemic affected schools in the current investigation. One study that focused on the writing proficiency of second-grade students found no significant effects of measures taken to mitigate the spread of the pandemic (Skar et al., 2023). Another investigation examining national test results in English, reading, and mathematics revealed either small or positive effects of the pandemic. Students in the COVID-19 cohorts scored as well as or better than students in previous cohorts (source: https://www.udir.no/tall-og-forskning/statistikk/analyser/mulige-konsekvenser-av-koronapandemien/resultater-pa-nasjonale-prover/). One possible explanation for these findings is that schools were only fully closed for approximately six weeks in the spring of 2020. During the 2021–2022 school year, decisions regarding remote instruction were made locally, and schools that did close for in-person instruction only did so for an average of three days (source: https://www.udir.no/tall-og-forskning/publikasjoner/utdanningsspeilet/utdanningsspeilet-2021/koronapandemien/). Therefore, while it cannot be completely ruled out that the pandemic disrupted the program's implementation, the available data suggest that student learning, in general, does not appear to have been significantly affected. Third, teachers were provided with professional development, but no on-going coaching was provided. It is possible that such coaching could have led to more positive outcomes.

With these limitations in mind, the primary finding from the current study was that increasing the amount of purposeful writing Grade 1 students did for a two-year period did not enhance the quality of their writing, handwriting fluency, and attitude towards writing when compared to typical writing practices. These findings did not support the efficacy of the *writing is caught* approach.

## Footnotes

1 There were only trivial changes in the magnitude of the regression coefficients and no changes in conclusions of statistical significance for any of the independent variables.

2 In Norway it is common for teachers to follow a single class for the first three or four years.
